# Deciphering dynamic interactions among multidimensional psychological motivations, academic performance, and sociocultural adjustment: The critical influence of excessive WeChat use

**DOI:** 10.1016/j.heliyon.2024.e32329

**Published:** 2024-06-03

**Authors:** Hua Pang, Wenxue Ke, Wanting Zhang

**Affiliations:** a School of New Media and Communication, Tianjin University, Tianjin, 300072, China; b School of Humanities and Arts, Tianjin University, Tianjin, 300072, China; c Department of Literature, Art and Media Studies, University of Constance, 78464, Constance, Germany

**Keywords:** Psychological motivations, Cross-cultural adaptation, WeChat, Academic performance, Sociocultural adjustment, International students

## Abstract

As the preeminent mobile social media platform in Mainland China, WeChat's meteoric expansion has revolutionized the dynamics of interpersonal communication and the modalities of mutual interaction among individuals. Despite the escalating significance of mobile social media in the cross-cultural adaptation of international students, existing scholarly works have largely neglected the underlying relationship between psychological motivations, excessive WeChat use, academic performance, and sociocultural adaptation of these students. Anchored in uses and gratifications theory and cross-cultural adaptation paradigm, the primary objectives of this study are to devise a theoretical model and to scrutinize differential psychological motivations propelling international students' WeChat usage, its association with excessive WeChat use, and impacts on academic performance and sociocultural adjustment. The proposed model undergoes meticulous evaluation through data amassed online from 598 Chinese international students studying in Germany. Sequential analytical techniques, especially Confirmatory Factor Analysis and Structural Equation Modeling, were employed to elucidate the dynamic interplay among key variables. The final results validate the path effect suggesting that both hedonic and social motivations are positive predictors of excessive WeChat use. Moreover, excessive WeChat use is negatively correlated with academic performance and sociocultural adjustment. However, utilitarian motivation is not significantly related to excessive WeChat use. By demystifying the driving factors and consequences of excessive WeChat use, these findings not only accentuate the pivotal role of mobile social media in the cross-cultural adaptation of international students but also enrich the theoretical landscape and enhance the strategic approaches for educators and academic institutions.

## 1 Introduction

Owing to the growing globalization of higher education in numerous developed nations, such as the USA, Australia, Canada, and Germany, it has been evident that the mobility of international students has also increased significantly during the past few years. Germany is renowned for its exemplary education system and a rich legacy of scientific research, which render it an alluring destination for international students seeking to achieve their academic aspirations [1]. Since 2011, China has been Germany's top source of international students, and the number has increased annually. During the winter semester of 2020/21, approximately 40,000 Chinese students were enrolled in German universities [2]. The increasing influx of international students is regarded as a significant cultural and economic contribution to the host country [3,4]. Universities nowadays operate within a context of heightened global competition for recruiting international students [5,6]. For universities that compete to attract international students, it is imperative to enhance the international students' overall experience and to assist them in coping with the stress of sociocultural adjustment and achieving their academic potential.

Recently, the usage of mobile Social Networking Sites (SNS) has become increasingly pervasive, providing individuals with a variety of opportunities to collaborate, capitalize, and share content that was previously inaccessible [[7], [8], [9]]. Prior research has consistently indicated that students consider mobile SNS as a practical medium to share their problems and seek solutions to general issues like social anxiety and loneliness [10,11]. Students are also prone to seek assistance in matters like medical counseling, unfamiliar experiences, examinations, and study skills [8]. As mobile social media increasingly permeates the daily lives of users, the imperative for conducting nuanced research into the effects of mobile SNS grows more critical. Relevant research is especially significant for international students, since previous studies have shown that students' problematic usage of mobile SNS can generate adverse and detrimental effects. For instance, existing studies have noted the negative impacts derived from students’ frequent interactions with online social networking and the virtual world, involving excessive use and addiction, isolation in real life, and a sharp decline in academic performance [4,9,10,12]. However, there still exist research gaps in the following three aspects.

Firstly, despite the burgeoning interest in understanding the phenomenon of excessive WeChat use among international students, there remains a notable dearth of comprehensive investigations into the underlying psychological motivations driving such behavior. Existing literature predominantly focuses on the prevalence and consequences of excessive mobile SNS use [5,13,14], yet fails to provide an in-depth exploration of the specific psychological factors influencing WeChat usage patterns among this demographic. Consequently, there exists a conspicuous gap in scholarly inquiry concerning the nuanced interplay between psychological motivations and excessive WeChat use among international students, necessitating further empirical investigation to elucidate this phenomenon.

Secondly, from an educational point of view, mobile SNS adoption and psychological motivations driving cross-cultural adaptation have garnered significant academic attention [8,15,16]. However, existing literature tends to overlook the potential negative consequences of this phenomenon, particularly in the field of educational information system research. There is a noticeable gap in empirical studies examining the relationship between excessive use of mobile SNS and academic performance, with even less attention given to the adverse effects on sociocultural adjustment. Moreover, with the increasing prevalence of digital social connections supplanting real-life interactions, it becomes imperative to comprehensively understand the factors influencing international students' excessive WeChat use. This understanding is crucial for mitigating potential negative effects on their academic performance and sociocultural adjustment [13,17]. Accordingly, this research primarily focuses on international students, given their propensity for disproportionate mobile SNS usage [4,17,18]. Conducting this survey will offer higher education administrators valuable insights to foster a more dynamic and innovative academic environment. Additionally, it will lay a foundational basis for the future development of an effective analytical model pertaining to international students’ use of mobile social media.

Thirdly, while excessive mobile social media use has ascended to a paramount societal issue, it is still unclear whether it can serve as a mediating factor between psychological motivations and international students' academic achievement and sociocultural adjustment. Few investigations have specifically adopted the uses and gratifications theory and cross-cultural adaptation framework within the educational field to determine the detrimental impacts of excessive WeChat use on academic achievement and sociocultural adjustment of international students. This research gap underscores the need for further empirical research to investigate the mediating role of excessive WeChat use. Such research would contribute significantly to the comprehensive understanding of the complex interplay between international students’ psychological motivation, excessive WeChat use, academic performance, and sociocultural adjustment.

By addressing the aforementioned research gaps, the present study is poised to meticulously investigate the following questions, aiming to shed light on these underexplored dynamics.

RQ1 What psychological motivations contribute to excessive WeChat use by international students?
RQ2 How does excessive WeChat use affect the academic performance and sociocultural adjustment of international students?
RQ3 Does excessive WeChat use mediate the correlation between psychological motivations and international students' academic performance and sociocultural adjustment?

Employing the theoretical foundations of uses and gratifications theory coupled with the cross-cultural adaptation framework, this study aims to bridge existing gaps in scholarly literature. Specifically, the study constructs a conceptual research model and establishes corresponding assumptions through a psychological perspective. Furthermore, the research meticulously explores the mediating effects of excessive WeChat usage. To validate this model, cross-sectional survey data from international students in Germany were collected and rigorously analyzed using Structural Equation Modeling (SEM).

This article makes several significant contributions to the existing body of knowledge. Firstly, it extends the scope of research on the adverse impacts of new media communication technologies into the educational domain, with a focus on excessive WeChat usage. Secondly, it enriches the understanding of the uses and gratifications paradigm by elucidating the mechanisms through which psychological motivations affect academic performance and sociocultural adjustment of international students. Lastly, the findings provide a foundation upon which scholars and practitioners can develop interventions or strategies to mitigate the potential negative repercussions of mobile social media on the well-being and behavioral outcomes of international students.

## 2 Theoretical framework and hypotheses development

### 2.1 Linking psychological motivations to excessive WeChat use

Over the past few years, numerous scholars have dedicated their efforts to investigating the diverse variables that determine the adoption or discontinuous decisions of mobile SNS platforms. These variables primarily encompass hedonic, utilitarian, and social motivation [6,19,20]. Hedonic motivation is closely associated with perceived amusement and enjoyment of SNS usage, making it a significant factor in the rising popularity of such newly emerging platforms among the younger generation [21]. Utilitarian motivation is conceptualized as individuals' perceptions of SNS services to enhance objective achievement correlated with interpersonal and information communication [6,21]. Finally, social motivation is correlated with perceptions of social relationships established, especially personal social position and prestige [6]. Excessive use is a term commonly employed to describe the extent to which mobile SNS usage exceeds the anticipated total time and energy requirements [22]. Limited research has explored individuals’ reflections on new media services that could assist in interpreting the degree of undesirable outcomes [5,9,22]. As mobile SNS platforms affect various aspects of daily life while also continuously developing special characteristics and functions, excessive use, or SNS addiction, is emerging as an important issue, especially among university students [8,23].

According to uses and gratifications theory, the interaction between mobile SNS usage and the perception of utilitarian, hedonic, and social motivation is complicated, with all three motivations serving as influencing factors of mobile SNS usage [19]. Prior research has conclusively demonstrated that all three identified motivations exert a significant effect on the excessive usage of diverse SNSs [6,24]. Combined with utilitarian variables, hedonic motivation has been demonstrated to be a dual construct in promoting mobile SNS platforms adoption, as hedonic motivation plays a crucial role in shaping individuals' attitudes toward mobile SNS platforms usage and is positively associated with their intention of continual usage [6,25]. The utilitarian value of SNS is linked to individuals’ capability to transmit information from a certain social media application. The enhancement of utilitarian variables provides improved commodities, productive capacity, and application [20,21]. Similar to hedonic motivation, utilitarian motivation exerts a positive and significant effect on the intention to continuously utilize mobile SNS [6,25].

Sociability involves opportunities for people to keep in touch with social groups, having an effect on self-expression, reception, identification, and demands [19,26]. Social motivation is a major variable in fostering connections establishment between individuals and their corresponding environment, as well as reconciling complicated interactions with environmental and interpersonal situations [26]. It provides opportunities for real-time interaction with people and groups across distances. Previous studies have asserted that a sense of social gratification may result in increased use of social media and could act as a vital indicator of the continuous use intention of SNS [6,27]. Similar to hedonic and utilitarian motivation, sociability motivation exerts a positive effect on the intention to continue using mobile social media. Based on the above analysis, this research hypothesizes.

H1 Hedonic motivation is positively related to excessive WeChat use.

H2 Utilitarian motivation is positively related to excessive WeChat use.

H3 Social motivation is positively related to excessive WeChat use.

### 2.2 Linking excessive WeChat use to academic performance

With the advancement of globalization, education for international students has emerged as a vital component of international academic exchange and collaboration. In this evolving academic landscape, international students are tasked with proactively adapting to novel academic systems and cultural environments to facilitate comprehensive development during their study abroad [3,28,29]. Concurrently, the prevalence of social media has provided additional opportunities for the development of international students. WeChat, as a mainstream mobile SNS, not only provides a convenient means of information dissemination but also acts as a linchpin for international students in maintaining social connections [[30], [31], [32]]. Nevertheless, excessive WeChat use may potentially disrupt the academic focus and depth of contemplation for international students [13,33]. In light of this context, this paper deems it necessary to explore the impact of excessive WeChat usage on international student education. Thus, it is imperative to contemplate the positioning of WeChat in academic development, aiming to better guide international students towards achieving superior academic performance and fully realizing their potential.

Generally, academic performance is associated with a student's ability to achieve a specific academic objective, which is commonly gauged by Grade Point Average (GPA) [13,23]. A multitude of scholars have explored the potential influences of using mobile SNS on students' academic performance [14,23,32,[34], [35], [36]], and some studies claimed positive effects [35,36]. Students can utilize mobile SNS to participate in social interactions through discussion and chat forums, apart from attending online conferences, online seminars, and accessing learning materials [35]. Moreover, mobile SNS also promotes constructive learning, which involves students and faculty collaborating to comprehend a certain subject, as opposed to a strategy that emphasizes individual contributions. Accordingly, students may have the opportunity to work as equal partners with faculty and classmates during the course of sharing opinions to enhance their academic performance [30].

In contrast to the potentially advantageous aspects, certain researchers have highlighted the negative effects of excessive mobile SNS usage on academic performance [13,37,38], with decreased performance being comparatively common. The far-reaching implications of excessive mobile SNS use have increasingly garnered considerable attention from various scholars over the past decades. For example, Junco found that excessive SNS use decreases the amount of time students spend studying and preparing for courses, which may negatively affect their GPA [11]. Likewise, Bou surveyed 112 undergraduate students in Lebanese and found a strong correlation between excessive SNS usage and poorer academic performance [39]. Moreover, Luqman et al. discovered that excessive social media use may lead to a loss of attention and distract students from completing their courses and assignments potentially resulting in non-educational and unproductive behaviors such as unnecessary conversations and random searches [40]. Furthermore, recent research extends this knowledge by identifying that users who overuse mobile social media achieve inferior academic performance and spend less than non-users [23,31].

The above-mentioned investigations reveal descriptive evidence of a negative relationship between excessive mobile SNS usage and academic performance. This article argues that excessive mobile social media use may interfere with the attention needed to achieve considerable academic achievement during the study period, such as sending text messages and posting status updates too frequently during class. Additionally, it can negatively influence students' mental concentration, which may distract students from their studies outside of school hours [22,33,34]. According to the cognitive load theoretical paradigm, the usage of mobile-based media in the classroom can simply overburden students' finite working memory and can also negatively affect deep learning [13]. Based on the above-mentioned discussion, the paper suggests that excessive WeChat use would weaken students’ mental resources and impair academic performance. Consequently, the current study hypothesizes as follows.

H4 Excessive WeChat use is negatively related to academic performance.

### 2.3 Linking excessive WeChat use to sociocultural adjustment

Typically, sociocultural adjustment is defined as the ability of a sojourner to cope with everyday life in the host country [17,28]. This adjustment is closely linked to individuals' social and practical competencies in integrating and negotiating the culture in an unfamiliar environment [28,32,41,42]. The degree of sojourners’ sociocultural adjustment is expressed in terms of the number of sociocultural challenges encountered and can be divided into challenges experienced in everyday life, academic research, and interpersonal relationships [16]. The accelerated development of globalization in education has led to a greater academic focus on the sociocultural adaptation of international students [28]. As a result of moving to a country with a different language and culture, the transitional experiences of international students may face unprecedented opportunities and challenges [3]. The transition from life ineluctably raises substantial stress factors (e.g. linguistic and cultural obstacles, loneliness, and academic pressures) which ultimately exert a negative impact on sociocultural adaptation [7,18]. Therefore, the establishment of new social networks in the host country is a crucial element of sociocultural adjustment [17].

Mobile social media has dramatically altered users' social relations and Internet behaviors, fostering interconnectedness and cooperation within society by shaping individuals’ social interaction [9,17].

An accumulating body of research has revealed that mobile SNSusage directly influences social networks, interpersonal communication, social integration, and social support among university students [4,29]. A considerable literature has demonstrated that wide-ranging and diversified mobile social networks are critical for enhancing international students’ adjustment [17,29]. Generally speaking, social networks provide individuals with numerous benefits, including extensive social resources, social support, and an overall sense of belonging [7]. Mobile social media may facilitate larger, more dispersed social networks, and enhance the contact between international students and local students [17].

Research examining the effects of excessive mobile SNS usage on sociocultural adjustment is finite, with available studies yielding conflicting outcomes. Some investigations suggested that international students use mobile SNSto interact with the community and obtain social support through online support groups [41]. Additionally, it has been posited that mobile social media usage is an essential condition for international students to enhance their social, cultural, and academic adaptation [7,17]. Conversely, a growing body of research has shown that excessive mobile social media use has a negative effect on sociocultural adjustment. Multiple empirical research has indicated a correlation between excessive mobile SNS usage and poor psychological well-being, including loneliness, depressive symptomatology, and social anxiety [10,23,43]. For instance, Wang's research suggested a curvilinear relationship between excessive Facebook use and prolonged feelings of loneliness [43]. Yoon's study also confirmed a weakly positive correlation between the time and frequency of SNS usage and depressive disorder [12]. Similarly, Keles et al. demonstrated that higher intensity of mobile SNS use is positively related to anxiety and psychological pressures [44]. Furthermore, excessive mobile social media usage tends to result in users spending less time on social activities [13]. Later, Youssef et al. claimed that excessive mobile social media usage could lead to a decline in an individual's social skills [45]. More recently, Shorter et al. discovered that mobile SNS use interferes with individuals' daily lives because of uncontrollable and excessive usage [15]. Therefore, the article raises the following hypotheses.

H5 Excessive WeChat use is negatively related to sociocultural adjustment.

## 3 Study methodology

### 3.1 Study model

**Fig. 1:**
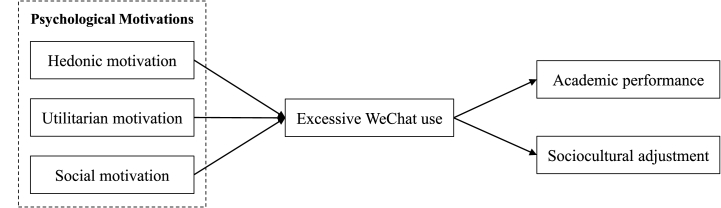
The conceptual research model.

Drawn on the preceding research, the article proposes a theoretical research model as shown in Fig. 1 and explained below. Building on past studies associated with excessive mobile SNS use [19,20,24,27], it is anticipated that utilitarian, hedonic, and social motivations of international students may contribute to the increase in their excessive WeChat use. This conceptual model investigates the extent to which three psychological motivations, hedonic, utilitarian, and social motivation, influence excessive WeChat use. Moreover, prior studies have explored various negative consequences of excessive mobile social media use [15,37,38,40]. The model thus hypothesizes that excessive WeChat use can adversely affect international students’ academic performance and sociocultural adjustment. The research model encompasses five pathways connecting excessive WeChat use to its motivations and consequences. The hypotheses that describe these pathways have been presented in the section above.

### 3.2 Sample and data collection

The survey targeted Chinese international students, a specific and potentially limited population at a comprehensive public university in Germany. The questionnaire for this survey was developed via a web-based data collection platform, viz., WJX.com, and circulated to the international students through an electronic link from November 18, 2022 to December 18, 2022. Based on ethical principles, this empirical Study was carried out with the approval of the Human Ethics Committee at the researchers’ college. All respondents were assured that their involvement with this investigation was voluntary and anonymous. Collected data would be kept confidential for academic purposes only. The questionnaire consists of seven sections: demographics, hedonic motivation, utilitarian motivation, social motivation, excessive WeChat use, academic performance, and sociocultural adjustment. The validity and reliability of this measurement model were evaluated before the formal distribution of the questionnaire. Table 1 shows the details of the measurement items. Once the questionnaires were collected, we cleaned the data, and 35 questionnaires were discarded for lack of response content. Despite the challenges associated with data collection from this unique group, we successfully obtained 598 effective responses, of which 351 were male (58.7 %) and 247 were female (41.3 %). Their ages ranged from 18 to 33 years old, with a major concentration in the 18 to 25 age group. In terms of WeChat usage and experience, the vast majority of participants (98.2 %) have been using WeChat for more than one year, and nearly half (46.8 %) have been using it for more than four years. Nearly half of the respondents (47.5 %) use WeChat for more than 4 h daily, and a very minority of respondents (11.9 %) use WeChat for less than 2 h daily. While the sample size may be considered relatively small in comparison to some larger-scale studies, we believe it provides a meaningful representation of the target population, given the specificity and constraints of the study context. Demographic characteristics of respondents are reported in Table 2.

**Table 1:** List of main variables and adapted constructs.

Variable	Item	Source
Utilitarian motivation	(1) I find WeChat very useful in my study abroad life. (2) I utilize WeChat to improve my productivity in the university. (3) I utilize WeChat to keep up with current events from friends. (4) I utilize WeChat to keep up with current events from mainstream news. (5) I utilize WeChat to stay informed about campus activities. (6) I utilize WeChat to enhance my performance in the university.	[6,46]
Hedonic motivation	(1) Engaging with WeChat brings me a great deal of pleasure. (2) Engaging with WeChat brings me a great deal of joy. (3) WeChat usage evokes feelings of enjoyment within me. (4) When using WeChat, I forget my daily worries. (5) When I'm bored, I use WeChat to have fun.	[25,47]
Social motivation	(1) I use WeChat to build favorable social bonds with others”. (2) I use WeChat to develop close friendships with other members within my social network. (3) I use WeChat to organize or take part in the diversified social event. (4) I use WeChat to maintain social connections with acquaintances and relatives that I don't normally have time to reach out to. (5) I use WeChat to make new friends. (6) I use WeChat to foster effective interpersonal communication.	[46]
Excessive WeChat use	(1) The time I spend on WeChat is always insufficient. (2) I spend an inordinate amount of time utilizing WeChat. (3) I spend much more time utilizing WeChat than most others. (4) I feel an increasing desire to use WeChat. (5) I always say ‘just a little longer’ when utilizing WeChat.	[5,10]
Academic performance	(1) I sometimes forget to attend classes. (2) My academic performance has gone downhill. (3) I have missed my course assignments. (4) I have faith in my academic capacities. (5) I feel proficient in completing my coursework.	[6,48]
Sociocultural adjustment	(1) I have adapted to the academic demands of the host university. (2) I have adapted to interacting with the locals. (3) I have understood the lifestyle of the host country. (4) I am really satisfied with my university environment. (5) I actively take part in various social activities in host university.	[17,29]

**Table 2:** Demographic characteristics of respondents (N = 598).

Categories	Frequency	Percentage (%)
Gender		
Male	351	58.7
Female	247	41.3
Age		
18–21 years old	166	27.8
22–25 years old	298	49.8
26–29 years old	97	16.2
30–33 years old	37	6.2
Educational level		
Undergraduate degree	358	59.9
Postgraduate degree	227	37.9
Doctoral degree	13	2.2
WeChat experience		
Below 1 year	10	1.8
1–2 years	36	6.0
2–3 years	98	16.4
3–4 years	173	28.9
Above 4 years	281	46.9
WeChat usage daily		
Below 1 h	20	3.4
1–2 h	51	8.5
2–3 h	81	13.5
3–4 h	162	27.1
Above 4 h	284	47.5

### 3.3 Measurement

The questionnaire for the survey contains two sections in total. The first section involves age, gender, monthly expenses, educational background, and WeChat using experience to gather participants’ socio-demographic data. The second section consists of six constructs, including utilitarian motivation, hedonic motivation, social motivation, excessive WeChat use, academic performance, and sociocultural adjustment. These constructs are measured using multi-item scales. To ensure content validity and tailor the research context of WeChat, all the measurement constructs used in the research were adapted and modified from previous research. To enhance the simplicity and intelligibility of this questionnaire, we recruited 30 international students who use WeChat to participate in a pilot test. Based on their feedback, some modifications were made to the questionnaire.

#### 3.3.1 Utilitarian motivation

Utilitarian motivation was gauged by six items modified from past studies [6,46]. The respondents were requested to indicate whether they agree with the given statements. The statements include: “I find WeChat very useful in my study abroad life”; “I utilize WeChat to improve my productivity in the university”; “I utilize WeChat to keep up with current events from friends”; “I utilize WeChat to keep up with current events from mainstream news”; “I utilize WeChat to stay informed about campus activities”; and “I utilize WeChat to enhance my performance in the university”. All the statements were developed according to a five-point Likert rating scale (1 = completely disagree, 5 = completely agree). Cronbach's alpha for this scale was 0.930. We calculated the average of the item scores to depict utilitarian motivation, with a higher score signifying a more pronounced level of utilitarian motivation.

#### 3.3.2 Hedonic motivation

Hedonic motivation scale was modified on the basis of previous studies [25,47]. Respondents were required to indicate the extent to which they agree or disagree with these items. Sample items conclude “Engaging with WeChat brings me a great deal of pleasure”; “Engaging with WeChat brings me a great deal of joy”; “WeChat usage evokes feelings of enjoyment within me”, “When using WeChat, I forget my daily worries”, and “When I'm bored, I use WeChat to have fun”. Cronbach's α coefficient for these items was 0.941. We averaged the scores to derive a hedonic motivation index. Higher scores on the scale indicate a stronger degree of hedonic motivation.

#### 3.3.3 Social motivation

Social motivation was reorganized from prior research [46] that measured social interaction and social engagement on WeChat. The participants were requested to indicate the extent to which they were keen to use WeChat “to build favorable social bonds with others”; “to develop close friendships with other members within my social network”; “to organize or take part in the diversified social event”; “to maintain social connections with acquaintances and relatives that I don't normally have time to reach out to”; “to make new friends”; and “to foster effective interpersonal communication”. The responses were based on a 5-point Likert scale ranging from 1 (completely disagree) to 5 (completely agree). Cronbach's alpha for these six items was 0.916. The scores on these six items were averaged to create an index of social motivation, with higher scores denoting a stronger level of social motivation.

#### 3.3.4 Excessive WeChat use

Excessive WeChat use was assessed by five items reorganized from existing research [5,10]. The chosen measurement was predicated upon its commendable validity and reliability, coupled with its capacity to evaluate the degree of excessive WeChat use. The items include “The time I spend on WeChat is always insufficient”; “I spend an inordinate amount of time utilizing WeChat”; “I spend much more time utilizing WeChat than most others”; “I feel an increasing desire to use WeChat”; and “I always say ‘just a little longer’ when utilizing WeChat”. Each item was gauged on a 5-point Likert scale where 1 = completely disagree and 5 = completely agree. Total scores were calculated to measure excessive WeChat use (Cronbach's α = 0.895). Thus, higher scores indicate a stronger degree of excessive WeChat use.

#### 3.3.5 Academic performance

Given the diverse evaluation criteria and methods for calculating GPA among distinct colleges and majors, it may be a complex and challenging task to utilize GPA as a sole indicator of academic performance. Therefore, the scale of academic performance was taken and revised from relevant literature [6,48]. The scale consists of five items: “I sometimes forget to attend classes”; “My academic performance has gone downhill”; “I have missed my course assignments”. The responses to these three questions were reversely coded for data analysis. “I have faith in my academic capacities”; “I feel proficient in completing my coursework”. A 5-point Likert scale was applied (1 “completely disagree” to 5 “completely agree”). The scores across five items were summed to generate a total score, reflecting academic performance. Higher scores on the scale indicates better academic performance.

#### 3.3.6 Sociocultural adjustment

The current study utilized the sociocultural adjustment scale developed by previous studies [17,29]. Items for the sociocultural adjustment scale consist of “I have adapted to the academic demands of the host university”; “I have adapted to interacting with the locals”; “I have understood the lifestyle of the host country”; “I am really satisfied with my university environment”; and “I actively take part in various social activities in host university”. The questionnaire options varied from 1 (completely disagree) to 5 (completely agree) based on a 5-point Likert scale (Cronbach alpha = 0.889). The scores on these items were summed to generate a total score representing sociocultural adjustment. Therefore, higher scores on the scale indicate a higher level of sociocultural adjustment among international students.

## 4 Data analysis strategy

Initially, an extensive data cleansing and pre-processing phase was undertaken using Microsoft Excel, a critical step to ensure the removal of any unqualified responses. Subsequently, this research incorporated the advanced analytical capabilities of SPSS version 24.0, utilizing it for both intricate descriptive statistical analysis and the evaluation of Common Method Variance (CMV), thereby establishing a foundational understanding of the data's characteristics and potential biases. Progressing to a more sophisticated level of analysis, the study employed Structural Equation Modeling (SEM) through the use of AMOS version 24.0. This choice was strategic, as SEM is renowned for its efficacy in testing and validating complex theoretical assumptions. Embracing a two-stage approach, as recommended by some scholars, the study first utilized Confirmatory Factor Analysis (CFA) to rigorously assess the proposed model [49]. This analysis encompassed an evaluation of the overall model fit, construct reliability, and validity, ensuring a comprehensive and robust assessment of the theoretical framework.

Upon confirming the model's validity, the study progressed to the second stage by employing SEM to validate the structural relationships posited within the model. The strength of SEM in this context lies in its confirmatory approach to survey data analysis, allowing for the precise identification of specific relationships between the main variables under study. Moreover, this approach enabled a profound exploration and elucidation of the intricate relationships within the dataset, offering valuable insights into the dynamics underpinning the study. Moreover, the deployment of SEM provided a critical advantage in assessing the factorial validity of the survey questions. This facet of the analysis is essential in determining the extent to which these questions accurately encapsulate the intended constructs and factors. By illuminating these intricate relationships, SEM facilitated a deeper and more nuanced understanding of the conceptual underpinnings of the study, thereby ensuring that the findings were not only statistically sound but also theoretically profound [50].

## 5 Results

### 5.1 Measurement model

The model is assessed on the basis of absolute fit indices (χ^2^/d.f. = 2.403; RMSEA = 0.018; RMR = 0.012) and the incremental fit indices (CFI = 0.951; AGFI = 0.967; IFI = 0.966; TLI = 0.946). These outcomes are presented in Table 3, which shows a satisfactory fit to the model. Cronbach's alpha and Composite Reliability (CR) are metrics used to demonstrate internal structure consistency. Data analysis shows that the whole Cronbach's alpha and CR values in this paper exceed the recommended threshold, verifying sufficient reliability. Likewise, convergent validity is determined through the examination of factor loadings, Average Variance Extracted (AVE), and Squared Multiple Correlations (SMC). The high loading on the variables testifies great convergent validity of the potential construct. The loading values ranged from 0.711 to 0.885, exceeding 0.7, indicating high convergent validity. The AVE of each construction is more than 0.5, which reveals appropriate convergence. All SMC values outstrip 0.5 implying convergent validity is established. The analytic outcomes concerning the confirmatory factor analysis are shown in Table 4. Some scholars state that AVE values would be contrasted with squared correlations of additional structures [51]. In Table 5, each AVE exceeds the related squared correlation coefficients signaling adequate discriminant validity. In summary, a measurement research model of the present study indicates an abundant model data fitting, high reliability, great convergent and discriminate validity.

**Table 3:** Fit indices for the measurement model.

Model fit measures	Model fit criterion	Index value	Good model fit (Y/N)
Absolute fit indices			
RMSEA	<0.08	0.018	Y
RMR	<0.05	0.012	Y
χ^2^/d.f. (χ^2^ = 187.461, d.f. = 78)	<3	2.403	Y
Incremental fit indices			
CFI	>0.9	0.951	Y
AGFI	>0.8	0.967	Y
IFI	>0.9	0.966	Y
TLI	>0.9	0.946	Y

**Table 4:** Statistical results of confirmatory factor analysis.

Constructs and items	Loading (>0.7)	SMC (>0.5)	CR (>0.7)	AVE (>0.5)
Utilitarian Motivation (UM)			0.932	0.696
UM1	0.785	0.616		
UM2	0.797	0.635		
UM3	0.846	0.716		
UM4	0.869	0.755		
UM5	0.877	0.769		
UM6	0.829	0.687		
Hedonic Motivation (HM)			0.939	0.757
HM1	0.879	0.773		
HM2	0.867	0.752		
HM3	0.858	0.736		
HM4	0.866	0.749		
HM5	0.881	0.776		
Social Motivation (SM)			0.913	0.637
SM1	0.795	0.632		
SM2	0.786	0.618		
SM3	0.787	0.619		
SM4	0.885	0.783		
SM5	0.747	0.558		
SM6	0.780	0.608		
Excessive WeChat Use (EW)			0.882	0.599
EW1	0.746	0.557		
EW2	0.802	0.643		
EW3	0.873	0.762		
EW4	0.728	0.529		
EW5	0.711	0.505		
Academic Performance (AP)			0.891	0.621
AP1	0.788	0.621		
AP2	0.799	0.638		
AP3	0.811	0.657		
AP4	0.814	0.663		
AP5	0.726	0.527		
Sociocultural Adjustment (SA)			0.886	0.609
SA1	0.763	0.582		
SA2	0.825	0.681		
SA3	0.761	0.579		
SA4	0.818	0.669		
SA5	0.729	0.531		

Notes: SMC, squared multiple correlations; CR, construct reliability; AVE, average variance extracted; UM, utilitarian motivation; HM, hedonic motivation; SM, social motivation; EW, excessive WeChat use; AP, academic performance; SA, sociocultural adjustment.

**Table 5:** Discriminant validity.

	UM	HM	SM	EW	AP	SA
UM	**0.834**					
HM	0.339**	**0.870**				
SM	0.415**	0.312**	**0.798**			
EW	0.262**	0.354	0.268**	**0.774**		
AP	0.243**	0.209**	0.345**	−0.421**	**0.788**	
SA	0.276**	0.266**	0.383**	−0.417**	0.322**	**0.780**

Notes: **p < 0.001. Diagonal elements (bold) represent the square root of AVE. Off-diagonal elements represent squared correlations between variables. UM, utilitarian motivation; HM, hedonic motivation; SM, social motivation; EW, excessive WeChat use; AP, academic performance; SA, sociocultural adjustment.

### 5.2 Common method bias

In this study, structured questionnaires were utilized to collect data from international students about the variables of interest. However, this approach raised concerns about Common Method Bias (CMB), a potential issue that could affect the integrity of the empirical data [52,53]. CMB is a type of systematic error in psychological or behavioral research, often associated with the data collection methods used [48,54]. Previous research has highlighted that CMB poses significant challenges, especially in self-report studies [48,53].

To address these concerns, the study implemented the Harman single-factor test, a methodological approach used to assess the extent of CMB. This involved conducting an Exploratory Factor Analysis (EFA) on all measured variables to ascertain whether a dominant factor accounted for a large portion of the covariance among them. The presence of such a factor would be indicative of substantial CMB [55]. However, the EFA results showed that the total variance for a single factor is less than 50 %, suggesting that CMB was not a significant issue in this research [54]. Further, the comparison between multivariate and univariate structures in the Confirmatory Factor Analysis (CFA) indicated that Common Method Variance (CMV) did not compromise the study's effectiveness.

In summary, the various tests employed to evaluate methodological variance indicated a minimal impact, leading to the conclusion that CMB was unlikely to have a significant effect on the research findings. This outcome enhances the credibility of the study's conclusions, affirming the reliability of the data collected through the structured questionnaires.

### 5.3 Structural model

**Fig. 2:**
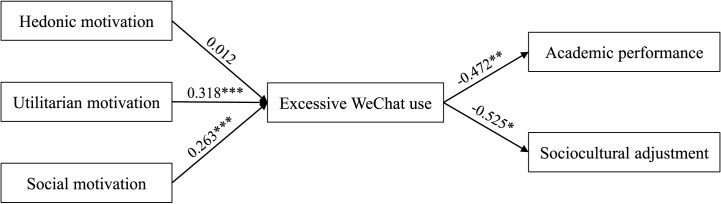
Outcomes for the structural equation model. *p < 0.05; **p < 0.01; ***p < 0.001.

Fig. 2 illustrates these outcomes for the structural equation model. This structural model is analyzed using AMOS 24.0, with ultimate model fit indices (χ^2^∕d.f. = 2.216 < 3; RMSEA = 0.015 < 0.08; RMR = 0.011 < 0.05; CFI = 0.966 > 0.9; AGFI = 0.978 > 0.8; IFI = 0.979 > 0.9; TLI = 0.968 > 0.9) indicating that the model fits well. After that, our article evaluates this structural model so as to identify these postulated connections. Analysis results demonstrate that the normalized pathway coefficients adequately confirm these assumptions statistically. Consistent with expectation, both hedonic motivation (β = 0.318, p < 0.001) and social motivation (β = 0.263, p < 0.001) positively predict excessive WeChat use. Hypothesis 2 and Hypothesis 3 are supported. Excessive WeChat use is negatively linked to academic performance (β = −0.472, p < 0.01) and sociocultural adjustment (β = −0.525, p < 0.05), statistically supporting H5 and H6. However, contrary to expectations, utilitarian motivation (β = 0.012, p > 0.05) is not associated with excessive WeChat use. Thus, Hypothesis 1 is rejected.

By utilizing bootstrapping analysis, the findings demonstrated that the relationship between utilitarian motivation and academic performance is partially mediated by excessive WeChat use (β = 0.122, p = 0.001 *<* 0.05; 95 % Confidence Interval (CI) = [0.053, 0.200]), the relationship between utilitarian motivation and sociocultural adjustment is partially mediated by excessive WeChat use (β = 0.081, p = 0.001 *<* 0.05; 95 % Confidence Interval (CI) = [0.067, 0.238]). Likewise, the relationship between social motivation and academic performance is partially mediated by excessive WeChat use (β = 0.117, p = 0.000 *<* 0.05; 95 % Confidence Interval (CI) = [0.065, 0.212]), the relationship between social motivation and sociocultural adjustment is partially mediated by excessive WeChat use (β = 0.079, p = 0.026 *<* 0.05; 95 % Confidence Interval (CI) = [0.011, 0.103]). The mediating effects study sheds light on the significance of utilitarian and social motivations, as these two factors can lead to excessive WeChat use, ultimately inducing numerous negative outcomes especially declined academic performance and lower level of sociocultural adjustment.

## 6 Discussion

### 6.1 Summary of key findings

The primary obstacle facing international students abroad is the need to achieve high academic performance while simultaneously adapting to social culture of the host country. This article conducts an empirical study on Chinese students pursuing their education in Germany, aiming to identify the underlying mechanism between psychological motivations, excessive WeChat use, academic performance, and cross-cultural adaptation. Employing a meticulously crafted survey questionnaire, the current study presents a hypothetical model linked to multi-dimensional psychological motivations and provides empirical evidence of its correlation with the academic performance and sociocultural adjustment of international students. This empirical research further examines the mediating role of excessive WeChat use. The findings of this investigation reveal the negative impact of excessive WeChat use among international students. Specifically, the research indicates that specific psychological motivations can prompt excessive use of WeChat, ultimately hindering their academic performance and sociocultural adjustment. Thus, obtained research consequences further communicate to relevant institutions the pressing need for valid intervention measures to alleviate the detrimental effects of excessive mobile social media use in the fields of education and cross-cultural adaptation.

First, unexpectedly, the finding reveals that hedonic motivation is not a significant factor in determining excessive WeChat use, contrary to past research findings, and hypothesis 1 is not valid. Different from open platforms (e.g., Weibo and TikTok), WeChat is a platform with higher privacy, which aims to facilitate communication and interaction among users' acquaintances [56]. Therefore, WeChat users typically have an extremely trustworthy online social network and tend to prioritize using WeChat for personal social interactions, rather than entertainment functions that other mobile SNSs (e.g., Weibo and TikTok) emphasize [56]. In summary, it is significant to note that diverse mobile SNSs have distinct features. WeChat is different from other mobile SNSs with respect to social context and functional localization, which means that the findings from other mobile SNSs should not be directly applied to the WeChat study. As a social tool, WeChat's main function is to facilitate interpersonal relationships and communication. Users are more inclined to use WeChat to maintain social networks, share life and practical information, rather than specifically seeking entertainment [8,56]. In addition, the use of WeChat is often closely linked to social connections in real life. This kind of real-world relevance may make users feel more restricted, as they are more focused on establishing and maintaining actual social relationships on WeChat, rather than simply pursuing entertainment [50]. Consequently, although hedonic motivation is an important motivation for social media use, social interaction and practicality may be more prominent in social tools like WeChat, thereby slowing down the trend of overuse.

Second, as confirmed by the significant result for H2, utilitarian motivation is positively linked to excessive WeChat use. Moreover, utilitarian motivation has a significant effect on excessive WeChat use. This outcome is in line with past research concerning mobile SNS [6,25]. As a platform for utility purposes, WeChat provides users with miscellaneous features, such as WeChat official account, circle of friends, and WeChat channels, which enable them to convey a diverse range of information to others conveniently. Besides, for WeChat users, the usage of WeChat is closely related to the perceived usefulness of the tool in performing tangible tasks, such as productivity management and content organization [6]. On the one hand, WeChat serves as an efficacious conduit for the fortification of interpersonal connections. Through WeChat, users can engage in real-time communication with friends, family, and colleagues, sharing their lives and thoughts. This convenient social interaction may lead some users to become addicted to the social functionalities of WeChat, excessively utilizing the platform to fulfill their social needs [21]. On the other hand, users can adopt WeChat to stay updated on others’ activities, share their own lives, and discover new friends. These attributes may encourage users to spend more time on WeChat, expanding their social circles in pursuit of broader social connections and a sense of belonging. Additionally, in certain situations, individuals may seek recognition and praise from others through social interactions on WeChat to enhance their self-worth and satisfaction [25]. When users receive positive feedback and praise from others on WeChat, they may experience a sense of fulfillment and pleasure, which may motivate them to use WeChat more to maintain this sense of satisfaction [6].

Third, the significant finding for H3 is consistent with previous studies, validating that social motivation is positively associated with excessive WeChat use. Compared to other motivations, social motivation is more enduring and interactional [46]. As a mobile social media, WeChat is an instrument for sustaining social support and enhancing interpersonal interaction, providing opportunities to engage with individuals and groups beyond time and space [50]. WeChat-based interaction can further extend users' social network, thus, users with strong social motivation may overuse WeChat to actively preserve social connections [20]. On the one hand, WeChat provides a convenient channel to strengthen interpersonal connections. Through WeChat, users can engage in real-time communication with friends, family, and colleagues, sharing their lives and thoughts. This convenient social interaction may lead some users to become addicted to the social functionalities of WeChat, excessively utilizing the platform to fulfill their social needs [10,46]. On the other hand, users can use WeChat to stay updated on others’ activities, share their own lives, and discover new friends. These features may encourage users to spend more time on WeChat, expanding their social circles in pursuit of broader social connections and a sense of belonging. Additionally, in certain situations, individuals may seek recognition and praise from others through social interactions on WeChat to enhance their self-worth and satisfaction [6]. When users receive positive feedback and praise from others on WeChat, they may experience a sense of fulfillment and pleasure, which may motivate them to use WeChat more to maintain this sense of satisfaction.

Fourth, as evidenced by the significant findings of Hypothesis 4, excessive WeChat use exhibits significant adverse effects on academic performance of international students, which is in accordance with existing studies. On the one hand, excessive use of WeChat can crowd out students' time outside of class and diminish their study time [13]. On the other hand, the excessive usage of WeChat to send messages and browse information during class may not only lead to a loss of attention [33] but may also overwhelm students’ limited memory capacity [13]. Furthermore, from a cognitive-emotional perspective, excessive social media usage may result in cognitive biases that can lead to privacy intrusion, technological burnout, and SNS fatigue, thus impacting the academic performance of international students who utilize mobile SNS excessively [5].

Lastly, the present research validates Hypothesis 5, suggesting that excessive WeChat use can impede sociocultural adjustment of international students. Past research has noted that excessive use of mobile SNS often leads to a decrease in the amount of time individuals spend on real-life social activities [11,31]. Additionally, excessive WeChat use may immerse users in the virtual world and reduce communication with those around them, resulting in a decrease in individuals’ social skills [45]. Therefore, it is crucial for international students to manage the diverse psychological motivations that affect excessive WeChat use. In addition, international students should be made aware of concerns correlated with excessive WeChat use and its potential adverse effects on academic performance and sociocultural adjustment.

### 6.2 Theoretical and managerial implications

Rooted in the above survey findings, the present study makes certain contributions to prospective research at the theoretical level. First, the majority of previous studies on the cross-cultural adaptation of international students have primarily focused on the impact of general mobile SNS use, while neglecting the diverse psychological motivations that may have totally distinct effects on academic performance and sociocultural adjustment [5,31,39]. Hence, through classifying WeChat use psychological motivations into three different dimensions: hedonic, utilitarian, and social motivation, this investigation launches a more systematic exploration of how the above-mentioned three specific psychological motivations of excessive WeChat use will affect academic performance and sociocultural adjustment of international students. By identifying the various influences of diverse WeChat usage psychological motivations, the article develops novel insights for the research areas of cross-cultural adaptation. Second, while existing research has provided valuable insights into the impact of mobile SNS usage on cross-cultural adaptation, there remains a dearth of exploration into the negative effects of excessive WeChat use on academic performance and sociocultural adjustment, which are integral to a comprehensive understanding of the phenomenon [4,17,39]. By developing an integrated conceptual model, the present research examines the repercussions of excessive WeChat use on academic performance and sociocultural adjustment. Understanding how excessive use of digital communication platforms influences academic outcomes and sociocultural adaptation contributes to the broader discourse on the role of technology in educational and cross-cultural contexts. Consequently, the results of the current research can also extend the concerned research and provide an original theoretical perspective for coming research on cross-cultural adaptation. Third, although former studies have determined the direct relationship between mobile SNS usage and cross-cultural adaptation, few investigations have been carried out to systematically probe into the internal mechanism among the factors [17,29]. Accordingly, the paper demonstrates that multi-dimensional psychological motivations and cross-cultural adaptation of international students are indirectly linked by the mediation of excessive WeChat use. Building on existing research, the findings of the current study deepen our comprehension of the mechanisms through which psychological motivations manifest in academic performance and socio-cultural adjustment. It provides a novel lens through which to view the intricate interplay between individuals’ psychological motivations, their utilization of mobile SNS, and the resulting impact on their educational and cultural experience.

In addition, in regard to managerial implications, the article provides practical recommendations for addressing issues pertaining to society, international students, and related institutions. Firstly, the article proposes that society as a whole ought to endeavor to foster a harmonious social atmosphere and a supportive academic environment to help international students adjust smoothly to university and complete their studies successfully. For example, relevant government departments can offer accommodative resources and appropriate psychological counseling services for international students. Secondly, it is emphasized that international students should appropriately utilize mobile social media. On the one hand, international students ought to excel in self-management. The current research indicates that multidimensional psychological motivations exert varying degrees of influence on excessive WeChat usage, among which utilitarian motivation has no correlation with excessive WeChat use. Consequently, international students are advised to proactively fulfill their utilitarian motivation by actively seeking information, sharing academic materials, and communicating with educators and peers via WeChat. On the other hand, the results of this study indicate that excessive WeChat use may adversely affect both academic performance and sociocultural adjustment. Therefore, international students are recommended to comprehensively comprehend the adverse outcomes associated with excessive WeChat use and conduct a thorough self-assessment of their self-control capabilities. For individuals who lack sufficient self-control, a proactive approach could involve installing time management applications on their mobile devices to restrict the time spent on mobile SNS. Finally, this paper emphasizes that universities should invest more effort and support. University administrators need to implement relevant interventions to promote proper mobile SNS usage. Specifically, they must reevaluate the necessity of using mobile social media in higher education settings. Simultaneously, educators should also convey the standards of mobile SNS usage and set up mechanisms to identify early signs of excessive usage. Furthermore, the relevant educational authorities should create an academic environment that promotes the multicultural integration of international students. Instructors need to consider the sociocultural backgrounds and previous experiences of multicultural students when implementing instructional strategies. Furthermore, counselors can provide international students with chances to acquire local language skills and engage with local people, thereby gradually enhancing their social self-efficacy.

### 6.3 Conclusion

Based on the empirical research, this study explores the dynamic interactions between multidimensional psychological motivations, academic performance, sociocultural adjustment, and excessive WeChat use among international students. While hedonic motivation does not exhibit a significant correlation with excessive WeChat use, the findings emphasize the substantial impact of utilitarian and social motivations on users' engagement with the platform. Specifically, utilitarian motivation displays a robust positive association with excessive WeChat use, highlighting the pragmatic considerations guiding users’ behaviors. Similarly, social motivation emerges as a significant predictor of excessive WeChat use, indicating the influence of social factors on online behaviors. Furthermore, the research reveals the detrimental consequences of excessive WeChat use on both academic performance and sociocultural adjustment among international students. These findings demonstrate the importance of developing interventions aimed at fostering responsible digital habits and facilitating the acculturation process within this demographic. In summary, the study contributes to a comprehensive understanding of motivations and negative effects of excessive WeChat use among international students. By identifying the pivotal roles of utilitarian and social motivations, the findings in this paper provide valuable insights for designing targeted interventions to mitigate the adverse effects of excessive technology use in this population.

## Limitations and implications for future research

While this research has provided implications for future studies to a certain extent, there remain some limitations that need to be discussed and addressed in future research. First, this paper explores only three specific psychological motivations underlying WeChat use. However, it is noteworthy that other psychological motivations for excessive WeChat usage remain unexplored and deserve further consideration in subsequent inquiries. It is advised that researchers can broaden the scope of their investigations and delve deeper into these overlooked psychological motivations to gain a more comprehensive understanding of WeChat usage. Second, this article concentrates on the diverse psychological motivations of WeChat usage, with a particular focus on the experiences of Chinese students studying in Germany. However, international students hailing from diverse countries and enrolled in varied educational settings might have varying psychological motivations for using social media. They may develop unique utilization patterns, giving rise to diverse usage behaviors. Considering the crucial role of social connectivity for international Asian students, future research could investigate the potential curvilinear impacts of WeChat usage, exploring how both low and high use might correlate with different outcomes. Moreover, personalized factors such as educational background, personal personality, and media literacy can impact users' motivation and behavior in using mobile SNS [[57], [58], [59]]. In light of the identified gaps, it is recommended that further studies should thoroughly consider the diversities in cultural backgrounds and study locations among international students, and incorporate personalized user characteristics into the survey, strengthening the applicability of the findings to a broader spectrum of the international student population. This analysis will also allow us to understand if our model operates similarly across different demographic or cultural groups, which is particularly pertinent given the international context of our study. Subsequent studies could conduct a multi-group analysis to assess the invariance of this model across different groups, thereby providing deeper insights into the generalizability of our findings. Third, this empirical research employs cross-sectional statistical analysis to investigate multi-dimensional psychological motivations and their effects on international students. Nevertheless, the method is limited in its capacity to elucidate causal relationships in the conceptual model. On the one hand, the dynamic nature of sociocultural adjustment and academic performance poses a challenge. On the other hand, mobile SNS use behavior of international students is perpetually shaped by environmental and cognitive factors, which will dynamically change in the long term. Given the constraints of AMOS in effectively addressing the non-normality of variables, future research could utilize more sophisticated statistical software capable of adeptly managing non-normal distributions. Therefore, this study recommends that future research could conduct longitudinal studies, experiments, and focus group method over a greater period of time to accurately assess how international students’ excessive WeChat use unconsciously influences sociocultural adjustment and academic performance. Fourth, although the participants were carefully selected to represent the target population of Chinese international students in Germany, the limited number of respondents may have restricted the generalizability of our findings. In future research, it is recommended to expand the sample size to include a more diverse and representative range of participants.

## Ethical statement and consent to participate

Approval was obtained from the ethics committee of Tianjin University with reference number: TJWHSX2302-01. Prior to data collection, written informed consent was obtained from study participants and actual data collection was conducted. The procedures used in this study adhere to the tenets of the Declaration of Helsinki.

## Funding

This research was funded by the Tianjin 10.13039/501100011788Philosophy and Social Science Planning Project (Grant No. TJWHSX2302-01).

## Data availability

The datasets used and/or analyzed during the current study are available from the corresponding author on reasonable request.

## CRediT authorship contribution statement

**Hua Pang:** Writing – review & editing, Writing – original draft, Investigation, Formal analysis, Conceptualization. **Wenxue Ke:** Writing – review & editing, Writing – original draft, Methodology, Investigation, Conceptualization. **Wanting Zhang:** Writing – review & editing, Methodology, Investigation, Funding acquisition.

## Declaration of competing interest

The authors declare the following financial interests/personal relationships which may be considered as potential competing interestsHua Pang is an Associate Editor for Heliyon and was not involved in the editorial review or the decision to publish this article. If there are other authors, they declare that they have no known competing financial interests or personal relationships that could have appeared to influence the work reported in this paper.

## References

[1] Bilecen B., Diekmann I., Faist T. (2023). Loneliness among Chinese international and local students in Germany: the role of student status, gender, and emotional support. Eur. J. High Educ..

[2] Li X., Primecz H. (2021). The future plans and dilemmas of Chinese students studying in Hungary: a narrative analysis. Soc. Econ..

[3] Yang P. (2022). China in the global field of international student mobility: an analysis of economic, human and symbolic capitals. Compare.

[4] Li X., Chen W. (2014). Facebook or Renren? A comparative study of social networking site use and social capital among Chinese international students in the United States. Comput. Hum. Behav..

[5] Cao X., Masood A., Luqman A., Ali A. (2018). Excessive use of mobile social networking sites and poor academic performance: Antecedents and consequences from stressor-strain-outcome perspective. Comput. Hum. Behav..

[6] Benson V., Hand C., Hartshorne R. (2019). How compulsive use of social media affects performance: insights from the UK by purpose of use. Behav. Inf. Technol..

[7] Cao C., Meng Q. (2020). Effects of online and direct contact on Chinese international students' social capital in intercultural networks: testing moderation of direct contact and mediation of global competence. High Educ..

[8] Hosen M., Ogbeibu S., Giridharan B., Cham T.-H., Lim W.M., Paul J. (2021). Individual motivation and social media influence on student knowledge sharing and learning performance: evidence from an emerging economy. Comput. Educ..

[9] Kim J.-h., Jung S.-h., Choi H.-j. (2023). Antecedents influencing SNS addiction and exhaustion (fatigue syndrome): focusing on six countries. Behav. Inf. Technol..

[10] Gao Q., Li Y., Zhu Z., Fu E., Bu X., Peng S., Xiang Y. (2021). What links to psychological needs satisfaction and excessive WeChat use? The mediating role of anxiety, depression and WeChat use intensity. BMC Psychology.

[11] Junco R. (2012). Too much face and not enough books: the relationship between multiple indices of Facebook use and academic performance. Comput. Hum. Behav..

[12] Yoon S., Kleinman M., Mertz J., Brannick M. (2019). Is social network site usage related to depression? A meta-analysis of Facebook–depression relations. J. Affect. Disord..

[13] Masood A., Luqman A., Feng Y., Ali A. (2020). Adverse consequences of excessive social networking site use on academic performance: explaining underlying mechanism from stress perspective. Comput. Hum. Behav..

[14] Ning W., Inan F.A. (2023). Impact of social media addiction on college students' academic performance: an interdisciplinary perspective. J. Res. Technol. Educ..

[15] Shorter P., Turner K., Mueller-Coyne J. (2022). Attachment Style's impact on loneliness and the motivations to use social media. Computers in Human Behavior Reports.

[16] Sarmiento A.V., Pérez M.V., Bustos C., Hidalgo J.P., del Solar J.I.V. (2019). Inclusion profile of theoretical frameworks on the study of sociocultural adaptation of international university students. Int. J. Intercult. Relat..

[17] Hassan Z., Jianxun C., Qaisar S., Shah Z., Ram M. (2021). Exploring the effect of WeChat on adjustment of international students in China. Cogent Psychology.

[18] Cao C., Zhu D.C., Meng Q. (2016). An exploratory study of inter-relationships of acculturative stressors among Chinese students from six European Union (EU) countries. Int. J. Intercult. Relat..

[19] Gan C., Li H. (2018). Understanding the effects of gratifications on the continuance intention to use WeChat in China: a perspective on uses and gratifications. Comput. Hum. Behav..

[20] Gong M., Yu L., Luqman A. (2020). Understanding the formation mechanism of mobile social networking site addiction: evidence from WeChat users. Behav. Inf. Technol..

[21] Anderson K.C., Knight D.K., Pookulangara S., Josiam B. (2014). Influence of hedonic and utilitarian motivations on retailer loyalty and purchase intention: a facebook perspective. J. Retailing Consum. Serv..

[22] Caplan S.E., High A.C. (2006). Beyond excessive use: the interaction between cognitive and behavioral symptoms of problematic Internet use. Commun. Res. Rep..

[23] Foroughi B., Griffiths M.D., Iranmanesh M., Salamzadeh Y. (2022). Associations between Instagram addiction, academic performance, social anxiety, depression, and life satisfaction among university students. Int. J. Ment. Health Addiction.

[24] Turel O., Serenko A. (2012). The benefits and dangers of enjoyment with social networking websites. Eur. J. Inf. Syst..

[25] Akdim K., Casaló L.V., Flavián C. (2022). The role of utilitarian and hedonic aspects in the continuance intention to use social mobile apps. J. Retailing Consum. Serv..

[26] Abplanalp S.J., Mote J., Uhlman A.C., Weizenbaum E., Alvi T., Tabak B.A., Fulford D. (2022). Parsing social motivation: development and validation of a self-report measure of social effort. J. Ment. Health.

[27] Arruda Filho E.J.M., Ferreira N.S. (2021). Technological usability in mobile networks: gratifications and risks related to using Whatsapp. Serv. Market. Q..

[28] Rhein D., Jones W. (2020). The impact of ethnicity on the sociocultural adjustment of international students in Thai higher education. Educ. Res. Pol. Pract..

[29] Cao C., Zhang J., Meng Q. (2022). A social cognitive model predicting international students' cross-cultural adjustment in China. Curr. Psychol..

[30] Astatke M., Weng C., Chen S. (2021). A literature review of the effects of social networking sites on secondary school students' academic achievement. Interact. Learn. Environ..

[31] Nti I.K., Akyeramfo-Sam S., Bediako-Kyeremeh B., Agyemang S. (2022). Prediction of social media effects on students' academic performance using Machine Learning Algorithms (MLAs). Journal of Computers in Education.

[32] Cao C., Meng Q., Zhang H. (2023). A longitudinal examination of WeChat usage intensity, behavioral engagement, and cross-cultural adjustment among international students in China. High Educ..

[33] Al-Busaidi A.S., Dauletova V., Al-Wahaibi I. (2022). The role of excessive social media content generation, attention seeking, and individual differences on the fear of missing out: a multiple mediation model. Behav. Inf. Technol..

[34] Marker C., Gnambs T., Appel M. (2018). Active on Facebook and failing at school? Meta-analytic findings on the relationship between online social networking activities and academic achievement. Educ. Psychol. Rev..

[35] Koranteng F.N., Wiafe I. (2019). Factors that promote knowledge sharing on academic social networking sites: an empirical study. Educ. Inf. Technol..

[36] Zhang X., Abbas J., Shahzad M.F., Shankar A., Ercisli S., Dobhal D.C. (2024). Association between social media use and students' academic performance through family bonding and collective learning: the moderating role of mental well-being. Educ. Inf. Technol..

[37] Tarafdar M., Darcy J., Turel O., Gupta A. (2015). The dark side of information technology. MIT Sloan Manag. Rev..

[38] Wu J.-Y., Cheng T. (2019). Who is better adapted in learning online within the personal learning environment? Relating gender differences in cognitive attention networks to digital distraction. Comput. Educ..

[39] Bou-Hamad I. (2020). The impact of social media usage and lifestyle habits on academic achievement: insights from a developing country context. Child. Youth Serv. Rev..

[40] Luqman A., Masood A., Shahzad F., Shahbaz M., Feng Y. (2021). Untangling the adverse effects of late-night usage of smartphone-based SNS among University students. Behav. Inf. Technol..

[41] Wong M.L.L., Liu S. (2024). The role of online social networking sites in facilitating friendships and adaptation among international students in Malaysia. Int. J. Intercult. Relat..

[42] Lai C., Cai S. (2023). The nature of social media use and ethnic minorities' acculturation. Int. J. Intercult. Relat..

[43] Wang K., Frison E., Eggermont S., Vandenbosch L. (2018). Active public Facebook use and adolescents' feelings of loneliness: evidence for a curvilinear relationship. J. Adolesc..

[44] Keles B., McCrae N., Grealish A. (2020). A systematic review: the influence of social media on depression, anxiety and psychological distress in adolescents. Int. J. Adolesc. Youth.

[45] Youssef L., Hallit R., Kheir N., Obeid S., Hallit S. (2020). Social media use disorder and loneliness: any association between the two? Results of a cross-sectional study among Lebanese adults. BMC Psychology.

[46] Wang G., Zhang W., Zeng R. (2019). WeChat use intensity and social support: the moderating effect of motivators for WeChat use. Comput. Hum. Behav..

[47] Suhail K., Bargees Z. (2006). Effects of excessive Internet use on undergraduate students in Pakistan. Cyberpsychol. Behav..

[48] Shi C., Yu L., Wang N., Cheng B., Cao X. (2020). Effects of social media overload on academic performance: a stressor–strain–outcome perspective. Asian J. Commun..

[49] Anderson J.C., Gerbing D.W. (1988). Structural equation modeling in practice: a review and recommended two-step approach. Psychol. Bull..

[50] Chen X., Ma J., Wei J., Yang S. (2021). The role of perceived integration in WeChat usages for seeking information and sharing comments: a social capital perspective. Inf. Manag..

[51] Fornell C., Larcker D.F. (1981). Evaluating structural equation models with unobservable variables and measurement error. J. Market. Res..

[52] Miller S., Menard P., Bourrie D., Sittig S. (2022). Integrating truth bias and elaboration likelihood to understand how political polarisation impacts disinformation engagement on social media. Inf. Syst. J..

[53] Zheng L., Huang B., Qiu H., Bai H. (2023). The role of social media followers' agency in influencer marketing: a study based on the heuristic–systematic model of information processing. Int. J. Advert..

[54] Hossain M.A., Sabani A., Bandyopadhyay A., Raman R., Goyal D., Dwivedi Y.K. (2023). Investigating the effect of social media fake news on consumer behavior: an empirical study with multiple moderations. J. Strat. Market..

[55] Chen A. (2019). From attachment to addiction: the mediating role of need satisfaction on social networking sites. Comput. Hum. Behav..

[56] Hou R., Han S., Wang K., Zhang C. (2021). To WeChat or to more chat during learning? The relationship between WeChat and learning from the perspective of university students. Educ. Inf. Technol..

[57] Vraga E.K., Tully M. (2021). News literacy, social media behaviors, and skepticism toward information on social media. Inf. Commun. Soc..

[58] Tilleul C. (2023). Young adults' social network practices and the development of their media literacy competences: a quantitative study. Inf. Commun. Soc..

[59] Tran‐Duong Q.H., Vo‐Thi N.T. (2023). The influence of social media literacy on student engagement in online learning. J. Comput. Assist. Learn..

